# The activity regulation of the mitotic centromere-associated kinesin by Polo-like kinase 1

**DOI:** 10.18632/oncotarget.2843

**Published:** 2014-12-02

**Authors:** Andreas Ritter, Mourad Sanhaji, Kerstin Steinhäuser, Susanne Roth, Frank Louwen, Juping Yuan

**Affiliations:** ^1^ Department of Gynecology and Obstetrics, School of Medicine, J. W. Goethe-University, Theodor-Stern-Kai 7, 60590 Frankfurt, Germany; ^2^ Present address: University Hospital Jena, Institute for Diagnostic and Interventional Radiology, Experimental Radiology, Erlanger Allee 101, 07747 Jena, Germany

**Keywords:** MCAK, Plk1, phosphorylation, chromosome alignment, spindle assembly

## Abstract

The mitotic centromere-associated kinesin (MCAK), a potent microtubule depolymerase, is involved in regulating microtubule dynamics. The activity and subcellular localization of MCAK are tightly regulated by key mitotic kinases, such as Polo-like kinase 1 (Plk1) by phosphorylating multiple residues in MCAK. Since Plk1 phosphorylates very often different residues of substrates at different stages, we have dissected individual phosphorylation of MCAK by Plk1 and characterized its function in more depth. We have recently shown that S621 in MCAK is the major phosphorylation site of Plk1, which is responsible for regulating MCAK's degradation by promoting the association of MCAK with APC/C^Cdc20^. In the present study, we have addressed another two residues phosphorylated by Plk1, namely S632/S633 in the C-terminus of MCAK. Our data suggest that Plk1 phosphorylates S632/S633 and regulates its catalytic activity in mitosis. This phosphorylation is required for proper spindle assembly during early phases of mitosis. The subsequent dephosphorylation of S632/S633 might be necessary to timely align the chromosomes onto the metaphase plate. Therefore, our studies suggest new mechanisms by which Plk1 regulates MCAK: the degradation of MCAK is controlled by Plk1 phosphorylation on S621, whereas its activity is modulated by Plk1 phosphorylation on S632/S633 in mitosis.

## INTRODUCTION

The kinesin-13 family members of microtubule (MT) depolymerizers play essential roles in controlling MT dynamics [1-3]. Unlike other kinesins, the members of the kinesin-13 family do not use the energy from ATP turnover to move directionally along MTs but, instead, depolymerize them by disassembling tubulin subunits from the polymer end [4,5]. This family is characterized by the localization of the conserved kinesin motor domain in the middle of the polypeptide [6]. Among the members of this family, the mitotic centromere-associated kinesin (MCAK), the best characterized member, is a potent microtubule depolymerase [6]. The activity as well as the subcellular localization of MCAK are precisely regulated by phosphorylation events that are executed by a number of important kinases, such as Aurora B, Aurora A, cyclin-dependent kinase 1 (Cdk1) and Polo-like kinase 1 (Plk1) in mitosis [7-12].

Plks are a family of serine/threonine kinases involved in the regulation of the cell cycle and proliferation [13-16]. Plk1 is the most thoroughly investigated member of this family and has been attracting enormous attention as a cancer therapeutic target [17-21]. Plk1 fulfills various functions that are indispensable for a smooth mitotic progression by phosphorylating many substrates, such as Pin1, cyclin B1 and sororin [22-25]. Accordingly, it is found to be present in several subcellular localizations, including centrosomes [26], kinetochores [27] and central spindle where it participates in cytokinesis [28,29]. Plk1 interacts with members of the kinesin-13 family. In collaboration with Aurora A, Plk1 regulates the activity and subcellular localization of Kif2a during mitosis [30]. Plk1 phosphorylates Kif2b on S204 and T125 at kinetochores modulating its localization and function [31]. Plk1 promotes the catalytic activity of MCAK by phosphorylating the five residues at its C-terminus, thus regulating MT-dynamics and improving the correction of kinetochore-MT error-attachments [12]. As Plk1 phosphorylates very often different substrates at different mitotic stages, we have dissected individual phosphorylation of MCAK by Plk1 and characterized its function in more depth. Our data suggest that S621 is the major phosphorylation site of Plk1 on MCAK, which is responsible for regulating MCAK's degradation by promoting the association of MCAK with APC/C^Cdc20^ [32]. In the present study, we have addressed another two residues phosphorylated by Plk1, namely S632/S633 in the C-terminal domain of MCAK, and explored the function of the phosphorylation of these residues in mitosis.

## RESULTS

### Plk1 interacts and phosphorylates MCAK

**Figure 1:**
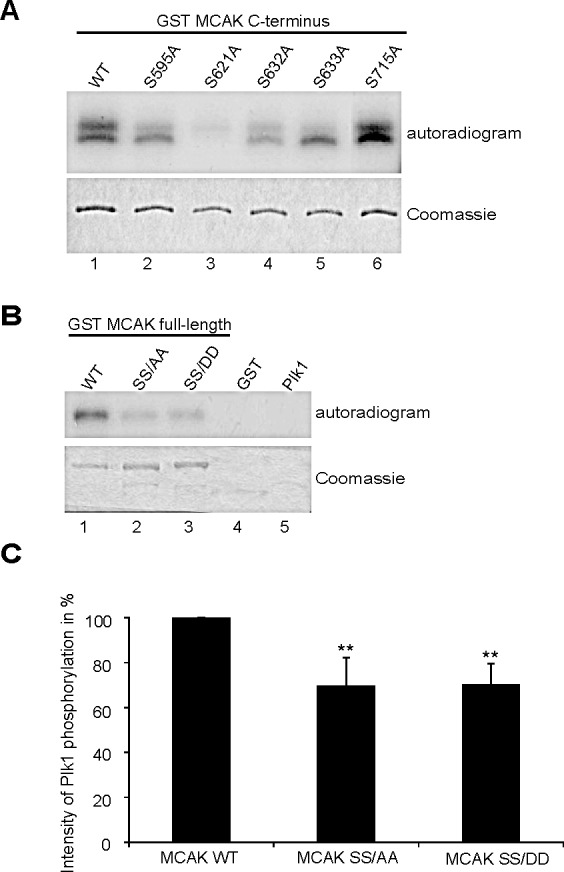
S632/S633 are phosphorylation sites for Plk1 (A) *In vitro* kinase assay of recombinant GST-MCAK C-terminus and its mutants. Phosphorylated MCAK C-termini by Plk1 were shown by autoradiography (upper panel). The gel was stained with Coomassie as loading control (lower panel). (B) *In vitro* kinase assay of recombinant full-length GST MCAK WT, GST MCAK SS/AA and GST MCAK SS/DD using Plk1 kinase. The assay was performed three times and one representative is shown. Upper panel: autoradiogram; lower panel: Coomassie staining as loading controls. (C) The quantification of Plk1 phosphorylation was performed using the Image J software, relative to the protein amounts from Coomassie staining. GST MCAK WT was assigned as 100%. The results are derived from three independent assays and presented as mean ± SEM. **p < 0.01.

Plk1 phosphorylates five serine residues at MCAK's C-terminus (S592, S595, S621, S633, and S715) modulating its catalytic activity [12]. To validate these Plk1 phosphorylation sites on the C-terminus of MCAK we performed an *in vitro* kinase assay using Plk1 and GST MCAK C-terminus fusion proteins carrying single alanine mutation of these putative sites. S621 was the major phosphorylation site of MCAK (Fig. 1A, lane 3), in line with our previous study [32]. Furthermore, mutation of S632 decreased the overall phosphorylation intensity and alteration of S633 led to loss of the upper phosphorylation band (Fig. 1A, lanes 4 and 5). While alanine substitution of S595 reduced the incorporation of labeled phosphate, mutation in S715 hardly changed the signal (Fig. 1A, lanes 2 and 6). Among the residues, S632, which does not fit the Plk1 phosphorylation consensus sequence and locates however directly in the front of S633, was obviously interfering with the phosphorylation (Fig. 1A). Thus, we decided to include it by introducing a double alanine or aspartic acid mutation into S632 and S633 (hereafter referred to as SS/AA and SS/DD, respectively) in the full-length GST MCAK. As illustrated in Fig. 1B and C, the double mutants reduced the phosphorylation signal by 30% relative to GST MCAK wild type. This finding shows that the S632/S633 residues are important phosphorylation sites for Plk1, beside the major phosphorylation site S621 in MCAK.

### Mimicking phosphorylation of S632/S633 by Plk1 increases the catalytic activity of MCAK *in vivo* and *in vitro*

**Figure 2:**
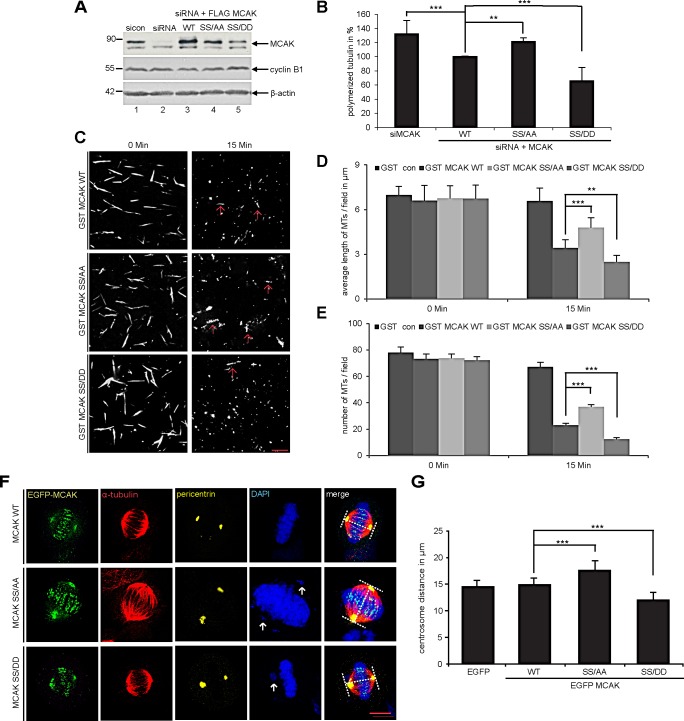
S632/S633 phosphorylation significantly enhances the microtubule depolymerizing activity of MCAK *in vivo* and *in vitro* Assessment of polymerized tubulin content in cells. HeLa cells were transfected with Flag-tagged MCAK WT, its mutants or the empty vector in an endogenous MCAK depleted background. Transfected cells were then synchronized to prometaphase and released for 1.5 h. (A) Western blot analysis with MCAK antibodies showing an efficient depletion of endogenous MCAK as well as the amount of each construct used in rescue experiment. β-actin served as loading control. (B) Cellular polymerized tubulin contents were analyzed by flow cytometry after cells were extracted, fixed and stained for α-tubulin. The amount of polymerized α-tubulin from Flag MCAK WT-transfected HeLa cells was assigned as 100%. The results are presented as mean ± SD (*n* = 3). ***p < 0.001, **p < 0.01. (C) *In vitro* depolymerization assay was performed as described in Materials and Methods. Example pictures at 0 and 15 min are shown. Red arrows indicate remaining faint microtubules. Scale bar: 10 μm. (D) Quantification of the microtubule length at indicated time points, measured with the AxioVision SE64 Rel. 4.9 software (n = 5 visual fields of 10.000 μm² for each condition). The results are presented as mean ± SD. **p < 0.01, ***p < 0.001. (E) Quantification of the number of microtubules at indicated time points (n = 5 visual fields of 10.000 μm² for each condition). The results are presented as mean ± SD. ***p < 0.001. (F) Measurement of spindle length. HeLa cells were transfected with EGFP-tagged MCAK WT, MCAK SS/AA, MCAK SS/DD or EGFP vector after depleting endogenous MCAK with siRNA. Cells were synchronized to the G2 phase using the Cdk1 inhibitor RO-3306 then released in the presence of MG132 for 2 h. Examples of metaphase cells rescued with EGFP MCAK and its mutant EGFP MCAK SS/AA and EGFP MCAK SS/DD are shown. White arrows indicate misaligned chromosomes. Scale bar: 7.5 μm. (G) The spindle length was measured using the LAS AF software (n = 30 metaphase cells for each condition). The results are presented as mean ± SD and statistically analyzed. ***p < 0.001.

To investigate the effect of S632/S633 phosphorylation by Plk1 on the catalytic activity of MCAK, we performed an *in vivo* microtubule depolymerization assay. HeLa cells depleted of endogenous MCAK were rescued with Flag-tagged MCAK WT, the non-phosphorylatable form MCAK SS/AA or the phosphomimetic mutant MCAK SS/DD. Figure 2A depicts the efficiency of MCAK depletion and the expressed amounts of MCAK WT and its mutants for rescue. HeLa cells expressing the non-phosphorylatable mutant MCAK SS/AA exhibited 22% more polymerized tubulin than in MCAK WT, comparable as HeLa cells depleted of MCAK (Fig. 2B). In contrast, cells rescued with the phosphomimetic mutant MCAK SS/DD displayed 34% less polymerized tubulin in relation to MCAK WT cells (Fig. 2B). These results could be also observed in HCT116 cells (Fig. S1A and B). Additionally, we carried out established depolymerization assays *in vitro*, as described [33]. Compared to wild type MCAK, MCAK SS/DD was found to be catalytically more active whereas MCAK SS/AA was more inactive to depolymerize stabilized microtubules (Fig. 2C), evidenced by evaluating the average length (Fig. 2D) and number (Fig. 2E) of remaining microtubules after 15 min reaction.

**Figure 3:**
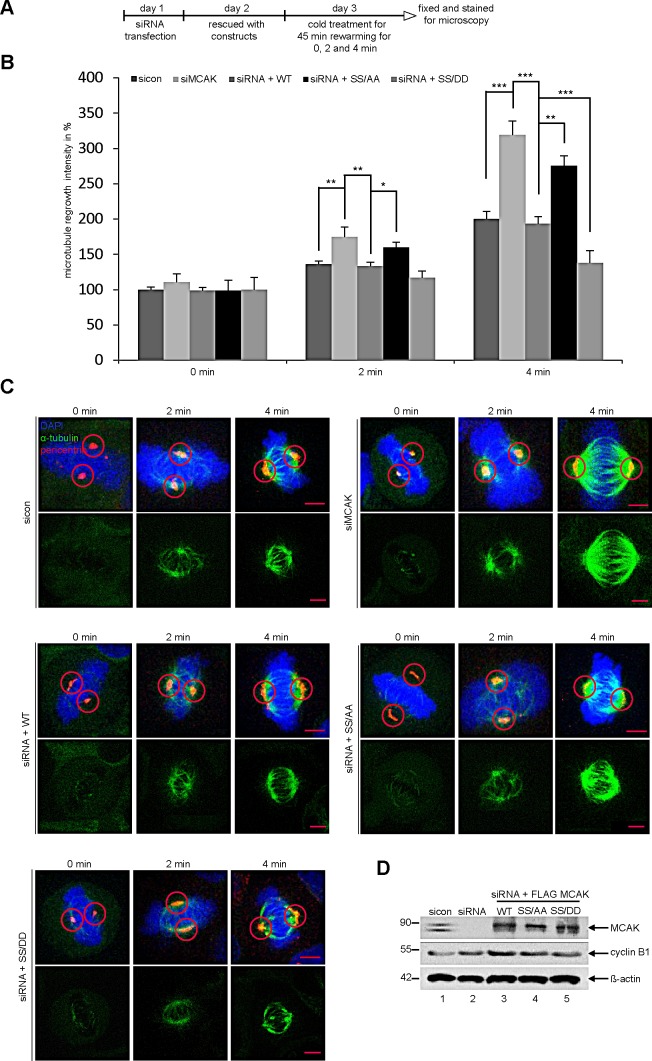
Reduced microtubules in cells rescued with MCAK SS/DD (A) Experimental schedule. HeLa cells depleted of endogenous MCAK were rescued with wild type MCAK or its mutants. Cells were cold-treated for 45 min and re-warmed for 0, 2 and 4 min. Cells were stained for α-tubulin, pericentrin and DNA. (B) Quantification of the microtubule amounts at indicated time points. Microtubule amounts in circled areas, shown in (C), were evaluated by confocal laser scanning microscopy with the LAS LF software (n = 30 metaphase cells for each condition). The results are presented as mean ± SD. *p < 0.05, **p < 0.01, ***p < 0.001. (C) Representatives are shown and circles indicate measured areas. Scale bar: 5 μm. (D) Western blot analysis for transfection's efficiency. β-actin served as loading control.

**Figure 4:**
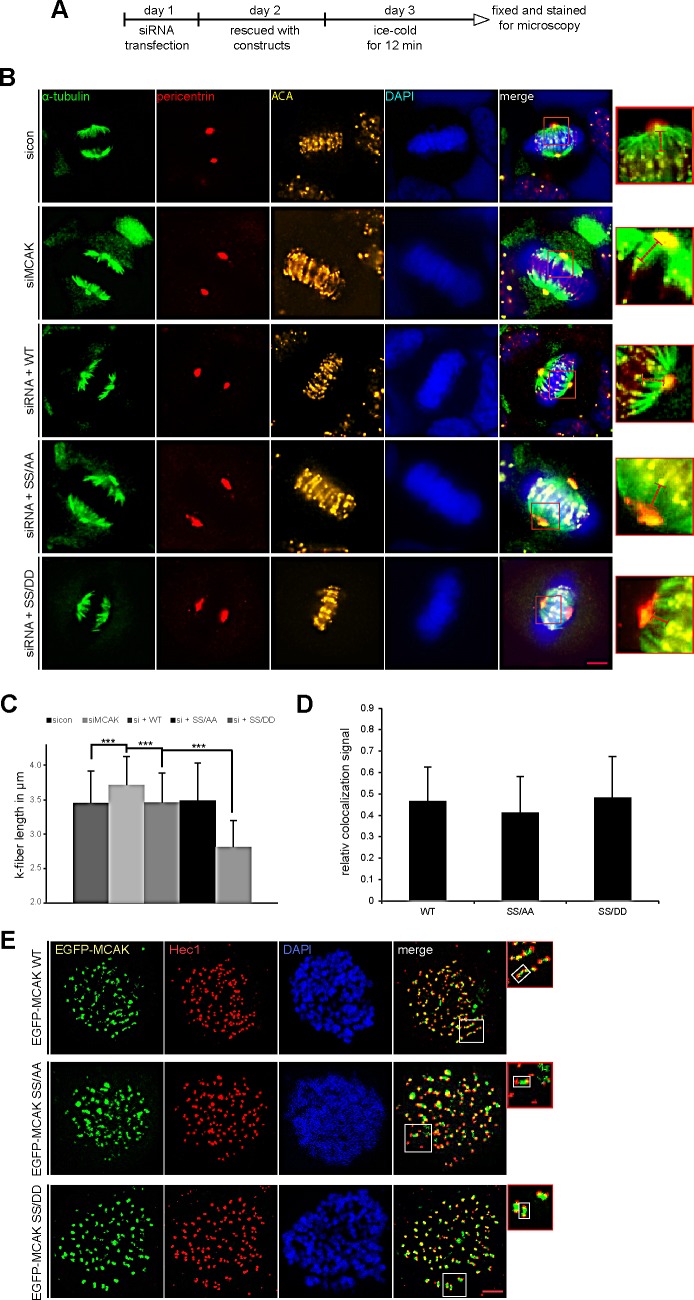
Decreased k-fiber length in cells with MCAK SS/DD and unchanged localization of MCAK mutants (A) Experimental schedule for B and C. HeLa cells depleted of endogenous MCAK were rescued with wild type MCAK or its mutants. Cells were ice-cold treated for 12 min and stained for α-tubulin, pericentrin, ACA (anti-centromere antibody) and DNA. (B) The k-fiber length was evaluated by fluorescence microscopy using the software AxioVision SE64 Rel. 4.9. (n = 80 fibers from 15 metaphase cells for each condition). The results are presented as mean ± SD. ***p < 0.001. (C) Representative cells, in which the k-fiber length was measured, are shown. Scale bar: 5 μm. Insets are magnified three fold and presented aside. Hardly change in kinetochore region of MCAK mutants. (D and E) Unchanged localization of MCAK S632/S633 mutants in the kinetochore region. HeLa cells depleted of endogenous MCAK were rescued with wild type EGFP-MCAK or its mutants. Cells were synchronized to prometaphase with nocodazol, released for 1 h into fresh medium containing MG132 to keep cells in metaphase. Metaphase cells were then exposed to hypotonic buffer, centrifuged onto an object slide and stained for Hec1 and DNA, as detailed in Materials and Methods. (D) Quantification of colocalized EGFP-MCAK constructs with Hec1 was performed using the Pearson's coefficient with ImageJ software. The results are presented as mean ± SD (n = 120 kinetochore pairs). (E) Representatives are shown. Insets are magnified two fold presented aside with a highlighted example of the kinetochore/centromere region. Scale bar: 2.5 μm.

To corroborate these observations, four further assays were performed. First, the spindle length was measured in cells transfected with EGFP MCAK WT, EGFP MCAK SS/AA and EGFP MCAK SS/DD. Like EFGP MCAK WT, EGFP MCAK SS/DD and EGFP MCAK SS/AA were localized in spindle poles and the kinetochore/centromere region (Fig. 2F, EGFP). Whereas HeLa cells expressing MCAK SS/AA showed significantly longer spindles, overexpression of MCAK SS/DD led to the formation of shorter spindles relative to cells transfected with EFGP MCAK WT, indicating a higher depolymerization activity of this phosphomimetic mutant (Fig. 2F and G). Similar results were obtained in U2OS cells (Fig. S1C and D). Second, a well-established microtubule regrowth assay [34] was performed. HeLa cells depleted of endogenous MCAK were re-added with Flag-tagged wild type or its mutants (Fig. 3D). Cells were then cold-treated for 45 min to depolymerize their microtubules, re-warmed at 37°C for 0, 2 and 4 min for re-polymerization, stained for α-tubulin, pericentrin and DNA. The areas depicted by the red circles in metaphase cells on Figure 3C represent the regions where the microtubule intensity was quantified as described elsewhere [34]. Relative to control groups, control siRNA treated or rescued with wild type MCAK, cells depleted of endogenous MCAK displayed increased amounts of microtubule upon rewarming (Fig. 3B and C). While intensified microtubule signals were observed in MCAK SS/AA transfected cells, less polymerized microtubules were assembled in MCAK SS/DD rescued cells (Fig. 3B and C). Third, the length of the k-fibers was measured as described [35]. For that, cells depleted of endogenous MCAK were rescued with either wild type MCAK or its mutants, cold-treated for 12 min, to depolymerize all other types of microtubules but k-fibers, and stained for microscopic evaluation (Fig. 4A). The metaphase cells rescued with MCAK SS632/633DD showed significantly shorter k-fibers whereas longer k-fibers were observed in cells depleted of endogenous MCAK or rescued with MCAK WT and MCAK SS/AA (Fig. 4B and C). Finally, the intensity of whole microtubules was measured in cells depleted of endogenous MCAK and rescued with wild type MCAK or its mutants. Again, cells expressing MCAK SS/AA had more microtubules whereas cells transfected with MCAK SS/DD exhibited less amounts relative to wild type MCAK rescued cells (Fig. S2), suggesting that these mutations indeed affect spindle microtubule polymers.

To exclude the possibility that phosphorylation could affect the subcellular localization of MCAK in the kinetochore region, where it functions, we performed a standard chromosome spreading assay [36]. HeLa cells were transfected with EGFP-tagged wild type MCAK or its variants, synchronized to metaphase and stained for DNA and Hec1, a kinetochore marker. Like wild type MCAK, both mutants localized in the inner region of Hecl staining (Fig. 4E). Further analysis of colocalization showed no significant difference between wild type MCAK and its mutants (Fig. 4D).

Collectively, the results from multiple experiments strengthen the suggestion that phosphorylation of S632/S633 by Plk1 promotes the depolymerizing activity of MCAK during mitosis.

### MCAK SS632/633AA retains cells in metaphase

**Figure 5:**
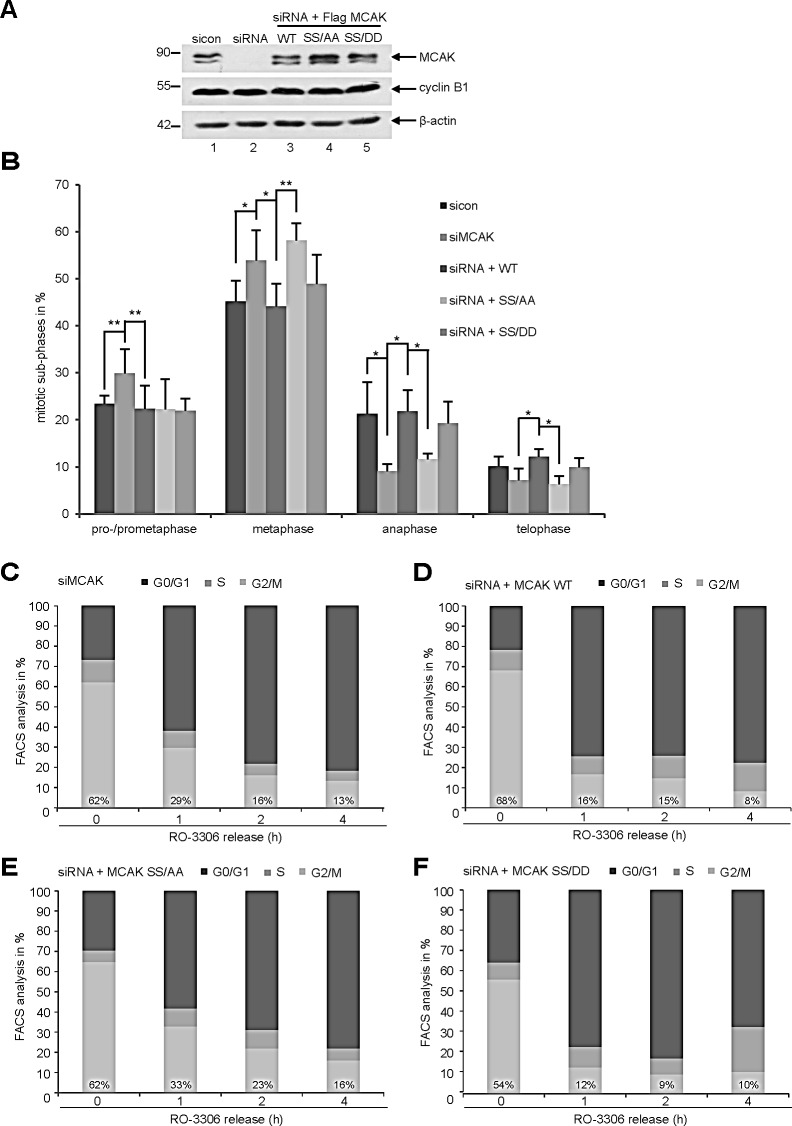
Interfering with MCAK SS632/633 phosphorylation causes a metaphase arrest Rescue experiment: HeLa cells were transfected with siRNA targeting only endogenous MCAK and followed by the rescue with Flag MCAK wild type and its mutants Flag MCAK SS/AA and Flag MCAK SS/DD. Cells were synchronized to the G2 phase with RO-3306 and released into fresh medium for 1.5 h. (A) Western blot analysis of the expression levels of Flag MCAK and its mutants in HeLa cells upon rescue. β-actin served as loading control. (B) Transfected HeLa cells were stained for α-tubulin and DNA and analyzed for distribution of the mitotic sub-phases using immunofluorescence microscopy. The results are displayed as mean ± SD. *p < 0.05, **p < 0.01. (C-F) HeLa cells, depleted of endogenous MCAK (C), rescued with MCAK WT (D), MCAK SS/AA (E) or with MCAK SS/DD (F), were synchronized to the G2 phase with RO-3306 and released into fresh medium. At indicated time points cells were harvested for cell cycle analysis by FACS.

To explore the functional significance of S632/S633 in mitosis, HeLa cells were knocked down of endogenous MCAK (Fig. 5A, 1^st^ row, lane 2) and replaced by Flag-tagged MCAK WT, MCAK SS/AA and MCAK SS/DD (Fig. 5A, 1^st^ row, lanes 3-5). Cells were synchronized to the G2-phase using the specific and reversible Cdk1 inhibitor RO-3306 [37] and released for 1 h to allow cells to complete mitosis. The distribution of mitotic sub-phases in different rescue groups was assessed using immunofluorescence microscopy. Knockdown of MCAK led to 30% of HeLa cells accumulated in pro- and prometaphase (Fig. 5B), confirming previous reports [38,39]. Expression of MCAK WT and its mutants could reverse this pro- and prometaphase arrest (Fig. 5B). Interestingly, cells expressing the non-phosphorylatable mutant SS/AA displayed difficulties in passing through metaphase by showing 14% more cells stuck in metaphase in relation to MCAK WT cells (Fig. 5B). MCAK SS/DD could decrease this arrest to 5%, slightly above the rate of cells rescued with MCAK WT (Fig. 5B). These data indicate that the non-phosphorylatable mutant SS/AA is not able to restore the function of endogenous MCAK in HeLa cells. Similar results were also observed in HCT116 cells (Fig. S3A and B). Further analysis with flow cytometry underscored the observation: after 1 h release from synchronization with RO-3306, about 30% of HeLa cells expressing MCAK SS/AA or HeLa cells depleted of MCAK still arrested at the G2/M phase (Fig. 5C and E), whereas cells rescued with MCAK WT or MCAK SS/DD went through mitosis and back to the G1-phase by showing only 16% and 12% at G2/M, respectively (Fig. 5D and F). The data indicate that knockdown of MCAK causes a transient delay in mitotic progression. Upon 4 h release, while 16% of MCAK SS/AA expressing cells were still kept at the G2/M phase (Fig. 5E), comparable to MCAK depleted cells (Fig. 5C), cells rescued with MCAK WT or MCAK SS/DD displayed only 8% and 10% at G2/M, respectively (Fig. 5D and F). This suggests that MCAK SS/AA expressing cells are not able to fully restore the function of endogenous MCAK and face problems by progressing through mitosis.

### MCAK S632/S633 mutants cause the formation of aberrant spindle and prevent proper chromosome alignment

**Figure 6:**
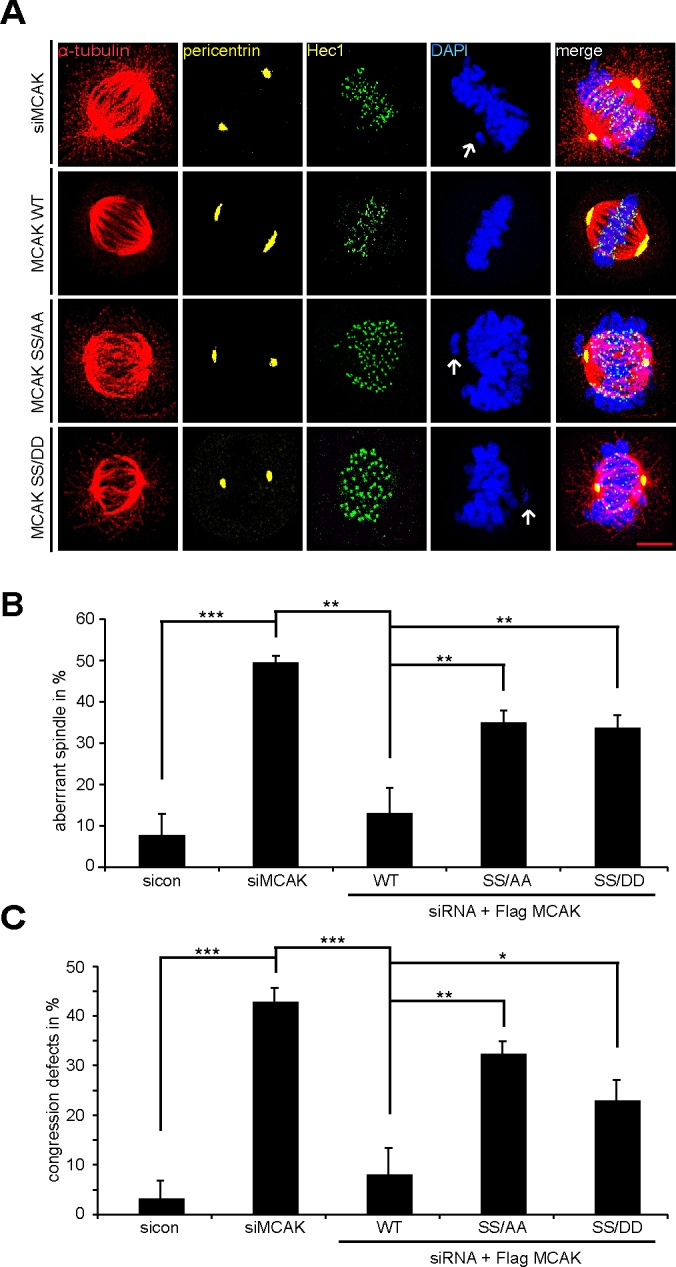
The mutants generate aberrant spindles and chromosome alignment failure Rescue experiment: HeLa cells were transfected with siRNA targeting only endogenous MCAK and followed by the rescue with Flag MCAK WT, Flag MCAK SS/AA or Flag MCAK SS/DD. Cells were synchronized to the G2 phase using RO-3306 and released into fresh medium for 1.5 h. (A) Cells were stained for α-tubulin, pericentrin, Hec1 and DNA. Representatives of aberrant spindles and chromosome fail-alignments are shown in HeLa cells transfected with siRNA against endogenous MCAK and rescued with Flag MCAK or its mutants. White arrows: misaligned chromosomes. Scale bar: 5 μm. (B) The percentages of cells showing defective aberrant spindles. The results are represented as mean ± SD and statistically analyzed. **p < 0.01. (C) The frequency of chromosome misalignment. The data are displayed as mean ± SD and statistically analyzed. *p < 0.05, **p < 0.01, ***p < 0.001.

We studied next the spindle morphology as well as chromosome positioning in cells depleted of endogenous MCAK and rescued 24 h later with Flag-tagged MCAK WT and its mutants. Treated cells were stained for MT marker α-tubulin, the kinetochore marker Hec1, the centrosome marker pericentrin and DAPI. 50% of MCAK knockdown cells exhibited the typical “hairy” spindles showing excessively long and aberrant tubulin nucleation (Fig. 6A, 1^st^ panel and B), in line with previous reports [38,40]. In addition, 43% of these cells failed to congress properly their chromosomes onto the metaphase plate (Fig. 6A, 1^st^ panel, white arrow, and C). Expression of MCAK WT reduced both spindle aberration and chromosome fail-alignment to respective 14% and 8% indicating the functionality of exogenous MCAK WT in depleted cells (Fig 6A, 2^nd^ panel, B and C). Interestingly, 35% of MCAK SS/AA cells displayed densely polymerized and aberrant spindles (Fig. 6A, 3^rd^ panel, tubulin staining, and B), whereas 30% of MCAK SS/DD cells demonstrated thin and less nucleated spindles (Fig. 6A. 4^th^ panel, tubulin staining, and B). In contrast to MCAK SS/AA expressing cells, in which 33% of metaphase cells showed chromosome congression failure (Fig. 6A, 3^rd^ panel and C), MCAK SS/DD could slightly reduce chromosome misalignments to 23% (Fig. 6A. 4^th^ panel, and C). Thus, neither MCAK SS/AA nor MCAK SS/DD could fully restore the phenotypes generated by depletion of endogenous MCAK. Comparable results were also obtained in HCT116 cells (Fig. S4A and B).

### The activity of MCAK S632/S633 mutants at the centromeres/kinetochores

**Figure 7:**
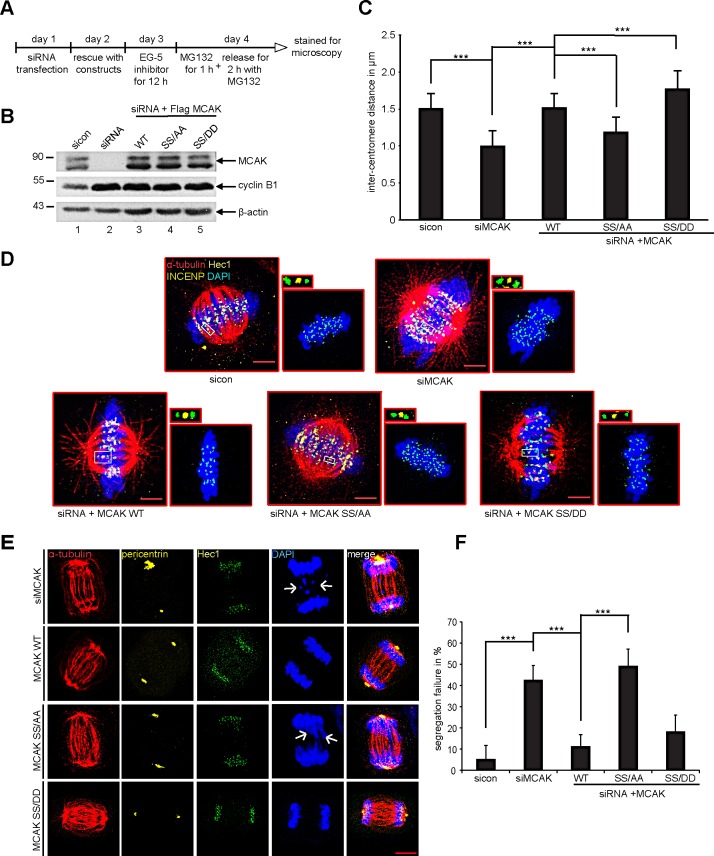
Expression of MCAK SS/AA reduces the inter-centromere distance and causes chromosome segregation defects The measurement of the inter-centromere distance in HeLa cells. (A) Working schedule. HeLa cells were transfected with Flag MCAK wild type, the non-phosphorylatable variant MCAK SS/AA, the phosphomimetic variant MCAK SS/DD after depletion of endogenous MCAK using siRNA targeting its 3′-untranslated region. Cells were synchronized to prometaphase using the Eg5 inhibitor III, the proteasome inhibitor MG132 was provided to the medium containing Eg5 inhibitor for the last hour, and cells were then released into fresh medium with MG132 for 2 h. (B) Western blot analysis of the expression levels of Flag MCAK and its mutants in HeLa cells upon rescue. β-actin served as loading control. (C) Treated cells were stained for α-tubulin, INCENP and Hec1 as centromere and kinetochore markers. The inter-centromere distance was measured using the LAS AF software (n = 90 kinetochore pairs for each condition). The results are presented as mean ± SD and statistically analyzed. ***p < 0.001. (D) Representative cells, in which the inter-centromere distance was measured. Scale bar: 7.5 μm. Paired centromeres with two fold magnification were also shown. (E) Representatives of mal-segregated chromosome in HeLa cells transfected with siRNA against endogenous MCAK and rescued with Flag MCAK or its mutants. White arrows: missegregated chromatids. Scale bar: 7.5 μm. (F) The frequency of cells showing missegregated chromosomes. The results are represented as mean ± SD and statistically analyzed. ***p < 0.001.

MCAK is involved in establishment of the tension between sister kinetochores [7,41]. To assess the local activity of MCAK S632/S633 mutants at centromere/kinetochore we measured the distance between sisters kinetochore in HeLa cells depleted of endogenous MCAK and rescued with either Flag-tagged MCAK WT, MCAK SS/AA or MCAK SS/DD. Cells were synchronized to prometaphase using Eg5 inhibitor III and released for 2 h in the presence of MG132 to prevent progression of cells beyond metaphase (Fig. 7A). Cells were stained for INCENP and Hec1, markers for centromeres and kinetochores. Endogenous MCAK was efficiently knocked down and the expression levels of transfected constructs were comparable (Fig. 7B). As expected, depletion of MCAK reduced the inter-centromere distance in HeLa cells and add-back of MCAK WT reestablished this distance (Fig. 7C and D). While the expression of MCAK SS/AA decreased the inter-centromere distance, suggesting a hampered catalytic activity of this mutant at the centrosome/kinetochore region, MCAK SS/DD increased significantly the centromere stretch in HeLa cells implying a higher depolymerization activity of this mutant relative to MCAK WT (Fig. 7C and D). Comparable results were also observed in HCT116 cells (Fig. S4C). These data underscore the observation derived from *in vivo* and *in vitro* depolymerization assay (Fig. 2-4) that the phosphorylation of S632/S633 by Plk1 activates MCAK during mitosis.

### MCAK SS632/633AA causes chromosome segregation defects

It is well established that MCAK is required to correct improper kinetochore-microtubule attachments during mitosis and interfering with this function leads to severe chromosome segregation defects [42]. Hence, we were next interested in the effect of MCAK mutants on chromosome segregation. To address this issue, HeLa cells depleted of endogenous MCAK were added back with wild type MCAK or its mutants and synchronized to mitosis. Depletion of MCAK increased dramatically chromosome segregation failure to 43% (Fig. 7E, 1^st^ panel, white arrow, and F) confirming the results of previous studies [2,42,43]. The introduction of ectopic MCAK WT as well as MCAK SS/DD reduced these defects to 11% and 18%, respectively (Fig. 7E, 2^nd^ and last panels, and F). Intriguingly, 49% of cells expressing the non-phosphorylatable form MCAK SS/AA were not able to properly segregate their chromosome and displayed lagging chromosomes (Fig. 7E, 3^rd^ panel, white arrows, and F). Similar results were obtained in HCT116 cells (Fig. S4D). These Data suggest that Plk1 phosphorylation of S632/S633 is required for correcting fail-attachments of the kinetochore-microtubules and preventing this phosphorylation leads to chromosome segregation defects in anaphase.

## DISCUSSION

It has been reported that Plk1 phosphorylates five residues of the C-terminal domain of MCAK and augments its catalytic activity in early mitosis [12]. To understand the precise regulation of MCAK by Plk1, it is crucial to decipher the function of each individual phosphorylation site in MCAK. In the present work, we have focused on S633, in association with S632, and have studied the functional effect of this double phosphorylation in mitosis.

We demonstrate that S632/S633 sites in the C-terminus of MCAK are targeted by Plk1 for phosphorylation by showing that the double non-phosphorylatable mutant MCAK SS/AA reduced the phosphorylation intensity by 30% (Fig. 1B and C). Unlike other mitotic kinases such as Aurora B and Cdk1 [9,11], Plk1 phosphorylates S632/S633 of MCAK and enhances its catalytic activity during mitosis, in support of a previous report [12]. Compared to MCAK WT expressing cells, cells rescued with MCAK SS632/633DD showed a decrease of polymerized tubulin *in vivo* (Fig. 2B and Fig. S1B) as well as *in vitro* (Fig. 2C-E), shorter spindles (Fig. 2F and F, and Fig. S1C and D) and shorter k-fiber after cold-treatment (Fig. 4B and C). Conversely, expression of the non-phosphorylatable mutant MCAK SS632/633AA generated longer mitotic spindles (Fig. 2F and G, and Fig. S1C and D) and an increased amount of polymerized tubulin *in vivo* (Fig. 2B and Fig. S1B) and *in vitro* as well (Fig. 2C-E). The data highlight that Plk1 regulates the catalytic activity of MCAK by phosphorylating S632/S633 at its C-terminus.

Furthermore, the phosphorylation of S632/S633 by Plk1 is essential for proper spindle assembly as well as for an accurate chromosome congression. Neither MCAK SS632/633AA nor MCAK SS632/633DD could rescue the assembly of aberrant spindles (Fig. 6A and B, and Fig. S4A). While MCAK SS632/633AA, the less active form, induced an increased regrowth rate (Fig. 3B) and densely polymerized tubulin (Fig. S2), the more active form MCAK SS632/633DD displayed a slower regrowth rate (Fig. 3B) and thin abnormal spindles (Fig. S2). These observations indicate that Plk1 phosphorylation on these sites affects the dynamics of microtubules by controlling MCAK's catalytic activity.

Depletion of centromeric MCAK causes a reduction of centromere stretch, alignment defects and severe missegregation of chromosomes [42,44]. Here, we found that none of these mutants could restore properly the chromosome alignment. Cells rescued with MCAK SS632/633AA or MCAK SS632/633DD exhibited significantly higher chromosomal misalignments relative to cells transfected with MCAK WT (Fig. 6C and Fig. S4B). These alignment failures could be directly ascribed to the deregulated activity of MCAK S632/S633 mutants at the centromere/kinetochore region, where an intact depolymerase activity is required to correct defective kinetochore-MT attachments [42]. This observation is further underscored by the measurement of the inter-centromere distance (Fig. 7C). Indeed, expression of both mutants generates abnormal inter-centromere stretch in cells. In parallel to its reduced catalytic activity (Fig. 2B-E and Fig. 3B), MCAK SS632/633AA induced a shorter inter-centromere distance similar to that in cells depleted of endogenous MCAK (Fig. 7C and D, and Fig. S4C). This implies that MCAK SS632/633AA is not able to effectively correct the defective kinetochore-microtubule attachments in these cells, which explains the increase in chromosome congression failure (Fig. 6C) and consequently the elevated chromatid segregation defect observed in these cells (Fig. 7E and F, and Fig. S4C and D). In contrast, the variant MCAK SS/DD generated an excessively high tension between sister kinetochores indicative of its hyperactivity in this region (Fig. 7C and D, and Fig. S4C). However, this high depolymerase activity could only slightly reduce the chromosome alignment failure in cells (Fig. 6A and C). Being abnormally elevated at centromere/kinetochore region, the enhanced activity of MCAK SS/DD will counteract the capture and stabilization of microtubules by the kinetochore, which will disturb the chromosome congression during early mitosis. In addition, alteration of microtubule dynamics within the spindle itself can also affect the inter-kinetochore tension. Collectively, this shows the importance of maintaining a proper activity of MCAK at a certain threshold during early phases of mitosis, allowing cells to nucleate microtubules, to form normal spindles and to properly attach and align their chromosome on the metaphase plate.

In summary, our data suggest that Plk1 phosphorylation of S632/S633 has to be spatially and temporally regulated. Our data underline the notion that this phosphorylation is required for proper spindle assembly during early phases of mitosis. The subsequent dephosphorylation of S632/S633, however, might be necessary to properly align the chromosomes onto the metaphase plate. This accurate regulation of chromosome congression and organization at metaphase is mandatory for a correct segregation of the chromatids at anaphase and prevents the loss of genetic material during cell division.

## MATERIALS AND METHODS

### Cell culture, synchronization, compound treatment and preparation of cellular extracts

HeLa, U2OS and HCT116 cells were grown according to supplier's instructions (DSMZ, Braunschweig). Cells were synchronized to the G1/S boundary with a double thymidine block, to the G2 phase with Cdk1 inhibitor RO-3306 [37] and to prometaphase with nocodazole treatment (50 ng/ml). To generate monopolar spindles cells were treated with Eg5 inhibitor III (1 μM, Sigma Aldrich) for 12 h followed by a release and treatment with the proteasome inhibitor MG 132 (10 μM, Sigma-Aldrich). Cell lysis was performed with RIPA buffer [11,45,46].

### Kinase assay *in vitro*

GST-tagged MCAK and C-terminus proteins were incubated with Plk1 kinase (Biomol, Hamburg) in the presence of 1 μCi [γ^32^P] ATP and 100 μM non-radioactive ATP at 37°C for 30 min. The reaction was stopped by adding sample buffer and boiling for 5 min. Equal volumes of each reaction were loaded onto 10% SDS PAGE and separated. The gel was stained with Coomassie and scanned as input control. The incorporation of ^32^P was quantified using Image J software.

### Western blot and cell cycle analysis

Western blot analysis was performed as described [47-49] and the following primary antibodies were used: mouse monoclonal anti-Kif2c (Santa Cruz biotechnology, Heidelberg), mouse monoclonal anti-Plk1 (Santa Cruz biotechnology), mouse monoclonal anti-cyclin B1 (Santa Cruz biotechnology), mouse monoclonal anti-β-actin (Sigma-Aldrich) and mouse monoclonal anti-Hec1 (Abcam, Darmstadt). Cell cycle was analyzed as described [17,19].

### siRNA knockdown and MCAK rescue experiments

siRNA targeting the 3′-untranslated region of MCAK was synthesized by Sigma-Aldrich. Control siRNA was purchased from Dharmacon Research Inc. (Lafayette). Cells were transfected with siRNA using the transfection reagent Oligofectamine (Invitrogen, CA) as described [48,49]. For phenotype analysis, HeLa cells depleted of endogenous MCAK were transfected with Flag-tagged MCAK WT or its mutants using FuGene-HD transfection reagent as instructed (Promega, Madisson). Treated cells were also used for cell cycle analysis, inter-centromere distance measurement, indirect immunofluorescence for evaluating chromosome positioning and spindle morphology. All experiments were independently performed at least three times.

### Immunofluorescence staining and measurement of inter-centromere and inter-centrosome distance

Immunofluorescence staining was performed as previously described [19]. Briefly, cells transfected with MCAK constructs were fixed for 8-10 min with methanol at −20°C or 4% paraformaldehyde containing 0.1% Triton X-100 for 15 min at room temperature. The following primary antibodies were used for staining: rat polyclonal antibody against α-tubulin (Biozol, Eching), rabbit antibody against pericentrin (Abcam), ACA, human anti-centromere antibody (Abcam), rabbit polyclonal antibody against INCENP (Abcam), mouse monoclonal antibody against Hec1 (Abcam) and mouse monoclonal antibody against Plk1 (Santa Cruz biotechnology). FITC-, Cy3- and Cy5 conjugated secondary antibodies were obtained from Jackson Immunoresearch (Newmarket). DNA was stained using DAPI (4′,6-diamidino-2-phenylindole-dihydrochloride) (Roche). Inter-centromere and inter-centrosome distance of metaphase cells were measured using the LAS AF software (Leica, Heidelberg). The immunofluorescence stained slides were further examined by a confocal laser-scanning microscope (CLSM) (Leica CTR 6500, Heidelberg). Images were processed using Adobe Photoshop.

### Construction of mutants and recombinant protein expression

Full-length human MCAK cDNA was obtained from RZPD (IRATp970F0111D, Berlin) and was cloned into BamH1/EcoR1 sites of pGEX 5x-3 (GE healthcare, Munich), into EcoR1/BamH1 sites of p3xFLAG-CMV7.1 (Invitrogen) and into BamH1/EcoR1 sites of pEGFP-C2 (Invitrogen). The C-terminus domain (aa 591-725) of MCAK was also sub-cloned into the pGEX 5x-3 using following primers: up primer: 5′-gggcccagtggagagcagttgatt-3′, down primer: 5′-tcactggggccgtttcttgctgcttat-3′. Point mutations were generated using the Quick Change-site-directed mutagenesis Kit (Stratagene, Amsterdam) using the following primers: SS/AA up: 5′-cgctgattccaggcaatttagccaaggaagagga-3, SS/AA down: 5′-tcctcttccttggctaaattgcctggaatcagcg-3; SS/DD up: 5′-ggaggaactgtcttcccagatggacgactttaacgaagccatgactcag-3′, SS/DD down: 5′-ctgagtcatggcttcgttaaagtcgtccatctgggaagacagttcctcc-3. Recombinant MCAK proteins were induced and expressed in *Escherichia coli* BL21 (DE3, *Codon Plus*) at 37°C for 2 h by addition of 1 mM IPTG and purified using glutathione-Sepharose 4B beads (GE Healthcare) as described previously [50].

### Measurements of the microtubule depolymerase activity *in vivo*

HeLa and HCT116 cells were depleted of endogenous MCAK using siRNA targeting the 3′-untranslated region of MCAK on day 1 and Flag-tagged MCAK plasmids were added back on day 2. Cells were synchronized with nocodazole and mitotic fraction was collected by shake-off. Mitotic cells were released for 1.5 h in fresh medium and collected for evaluating cellular microtubule polymer content after extraction, fixation and staining for tubulin using a FACSCalibur^TM^ (Becton Dickinson, Heidelberg), as described previously [39]. Briefly, cellular soluble tubulin was pre-extracted in a saponin-containing microtubule stabilizing buffer (2 mM EGTA, 5 mM MgCl_2_, 0.1 M PIPES pH 7.4, 0.2% saponin and 200 nM Paclitaxel). Resuspended cells in microtubule stabilizing buffer were then fixed with an equal volume of a 4% paraformaldehyde solution at 37°C for 15 min. Cells were then washed and stained for α-tubulin with specific mouse monoclonal antibody (Sigma-Aldrich) and FITC-conjugated rabbit anti-mouse antibody (DAKO, Jena), as described [39]. More than 95% of all cells were included in the acquisition gate and 100,000 cells were examined. Fluorescence intensity was quantified using the Cell Quest software (Becton Dickinson). Cells transfected with MCAK WT were assigned as microtubule polymer content 100%. The experiments were independently performed three times and each time was in triplicate.

### Microtubule depolymerization assay *in vitro*

The *in vitro* microtubule depolymerization assay was performed with modification as previously described [33]. In brief, x-rhodamine labeled bovine brain tubulin heterodimers (Cytoskeleton Inc, Biomol) were polymerized in G-PEM buffer (80 mM PIPES pH 6.9, 2 mM MgCl_2_, 0.5 mM EGTA, 1 mM dGTP and 10% glycerol) at 37°C for 20 min and then stabilized with 50 μM taxol for 5 min at 37°C. The assay was performed in a 50 μl reaction buffer containing 0.5 μl polymerized MTs, G-PEM buffer with 30% glycerol, 1 mM ATP, 20 μM taxol and 500 nM GST-MCAK or its variants for 15 min at 37°C. GST-tagged MCAK proteins were expressed and purified as described [11]. The reaction was terminated by addition of 200 μM taxol. After the reaction 5 μl of the reaction volume were immediately pipetted onto a glass slide, sealed for fluorescence microscopy, and evaluated with the AxioVision SE64 Re. 4.9 software.

### Metaphase chromosome spreading assay

HeLa cells were transfected with EGFP-tagged MCAK WT, MCAK SS/AA and MCAK SS/DD after depleting endogenous MCAK with siRNA. Cells were synchronized to prometaphase using nocodazole and released into fresh medium supplemented with the proteasome inhibitor MG132 to keep cells in metaphase. Metaphase cells were then subjected to hypotonic buffer (10 mM Hepes-Na pH 7.0, 30 mM glycerol, 1.0 mM Cal_2_ and 0.8 mM MgCl_2_) as described [36], centrifuged onto an object slide by using a cyto-centrifuge with 3000 RPM for 5 min, followed by fixation and stained for Hec1 and DNA. The chromosome spreads were further examined by a confocal laser-scanning microscope (Leica CTR 6500). The colocalization of EGFP-MCAK with Hec1 was quantified using the Pearsons's coefficient [51] and analyzed with the software ImageJ with the plugin Coloc2.

### Microtubule regrowth assay and k-fiber length measurement

This assay was performed with modifications as previously described [34]. In brief, coverslips with cells were incubated for 45 min in an ice bath to depolymerize microtubules. To allow microtubule regrowth, cells in coverslips were then incubated with warm medium at 37°C for 0, 2, and 4 min, followed by methanol fixation and stained for α-tubulin, pericentrin and DNA. The intensity of α-tubulin in a 4.06 μm diameter circle around centrosomes, as described [34], were evaluated by confocal laser-scanning microscopy using the LAS AF software (Leica). At least 30 cells per condition were measured. The k-fiber length was measured as described [35]. Briefly, cells were ice-cold treated for 12 min, fixed and stained for microscopy. The length of k-fibers was determined by measuring individual k-fiber from metaphase cells. For each condition, measurements were performed with 80 k-fibers of 15 independent cells. The k-fiber length was evaluated by fluorescence microscopy with the software AxioVision SE64 Rel. 4.9.

### Statistical analysis

Student's *t*-test (two tailed and paired) was used to evaluate the significance of difference between the transiently transfected cells with MCAK constructs. Difference was considered as statistically significant when p < 0.05.

## SUPPLEMENTARY MATERIAL FIGURES


