# Supplementary material for: The activity regulation of the mitotic centromere-associated kinesin by Polo-like kinase 1
Source: Oncotarget. 2014 Dec 2;6(9):6641–55. doi: 10.18632/oncotarget.2843 (PMC4466640; doi:10.18632/oncotarget.2843)
Supplement: Supplementary file 1 [file oncotarget-06-6641-s001.pdf]

The activity regulation of the mitotic centromere-associated kinesin by Polo-like kinase 1

Supplementary Material

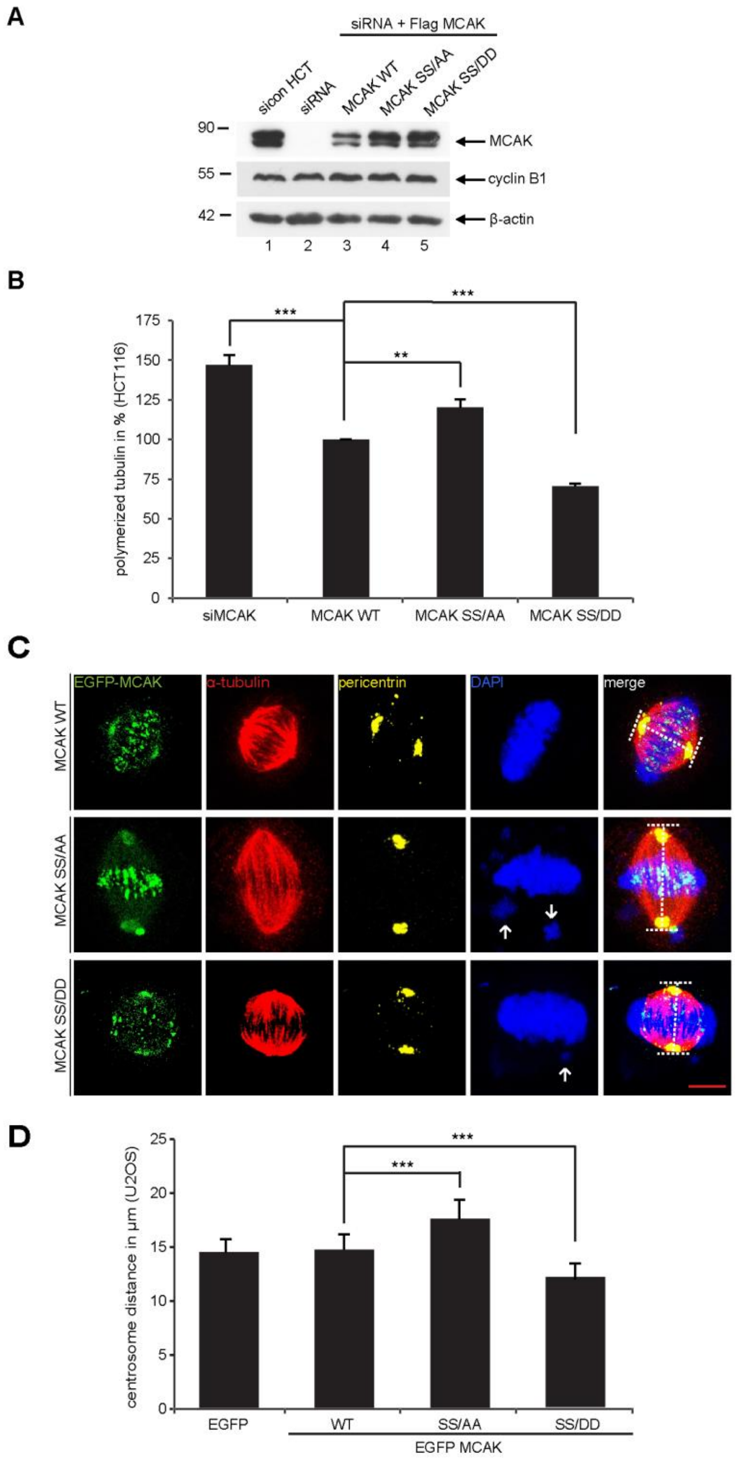

Figure S1: S632/S633 phosphorylation significantly affects its catalytic activity in colon cancer HCT116 cells and in osteosarcoma U2OS cells. HCT116 cells were transfected with

MCAK and its mutant MCAK SS/AA or MCAK SS/DD after depletion of endogenous MCAK. Cells were synchronized to prometaphase using nocodazole and released into fresh medium for 1.5 h. (A) Western blot carried out with MCAK antibodies showing the efficacy of MCAK depletion as well as the expression level of each construct used in rescue experiment. β-actin served as loading control. (B) Cellular polymerized α-tubulin contents were evaluated by flow cytometry after cells were extracted, fixed and stained for tubulin. The amount of polymerized tubulin from Flag MCAK WT-transfected HeLa cells was assigned as 100%. The results are presented as mean ± SD (n = 3). **$p < 0.01$, ***$p < 0.001$. (C) U2OS cells were transfected with EGFP-tagged MCAK WT, MCAK SS/AA, MCAK SS/DD or EGFP vector after depleting endogenous MCAK with siRNA. Cells were synchronized to the G2 phase using the Cdk1 inhibitor RO-3306 and then released in the presence of MG132 for 2 h. Examples of metaphase cells rescued with EGFP MCAK or its mutants are shown. Scale bar: 7.5 µm. (D) Measurement of the spindle length in U2OS cells. The inter-centrosome distance was measured using the LAS AF software. The results are presented as mean ± SD and statistically analyzed. ***$p < 0.001$.

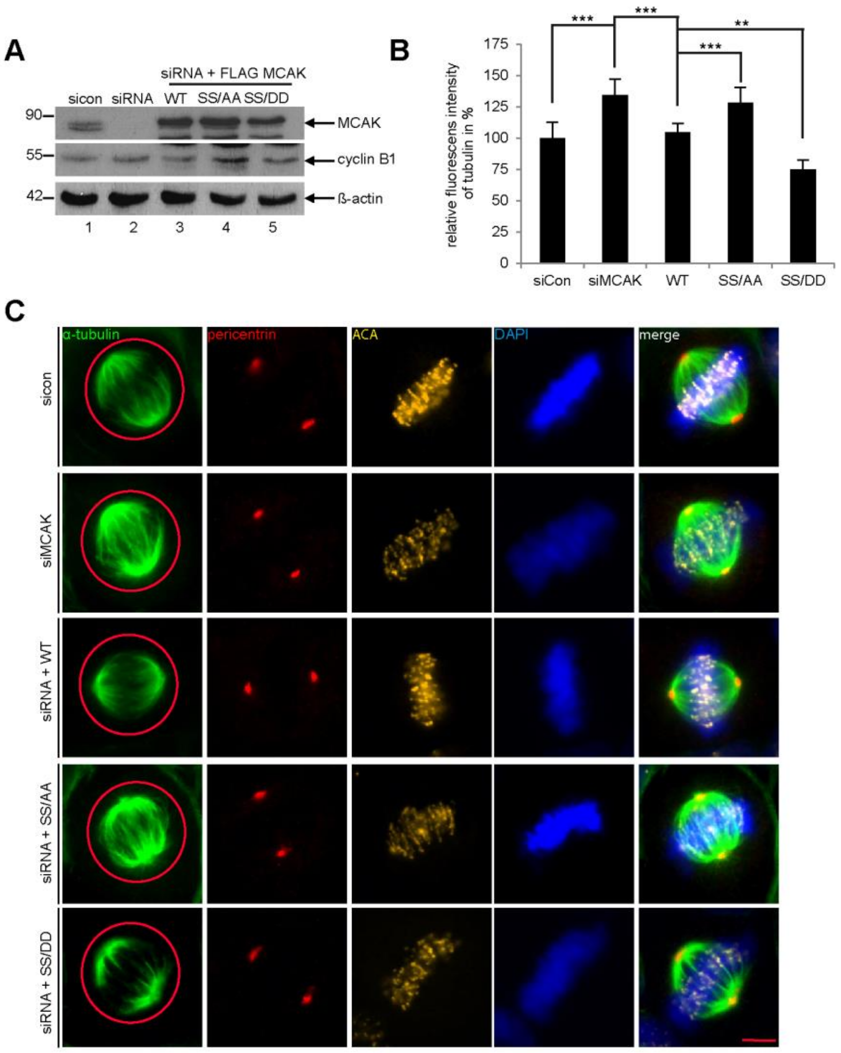

Figure S2: The amounts of whole microtubule are changed in cells expressing MCAK mutants. HeLa cells depleted of endogenous MCAK were rescued with Flag-tagged wild type MCAK or its mutants. Cells were synchronized to mitosis and stained for α-tubulin, pericentrin, ACA and DNA. (A) Western blot analysis for transfection's efficiency. β-actin served as loading control. (B) Quantification of whole microtubules of metaphase cells in circled areas shown in (C) with the LAS LF software (n = 30 metaphase cells for each condition). The results are presented as mean ± SD. $^{**}p < 0.01$, $^{***}p < 0.001$. (C) Representatives are shown and circles indicate measured areas. Scale bar: 5 µm.

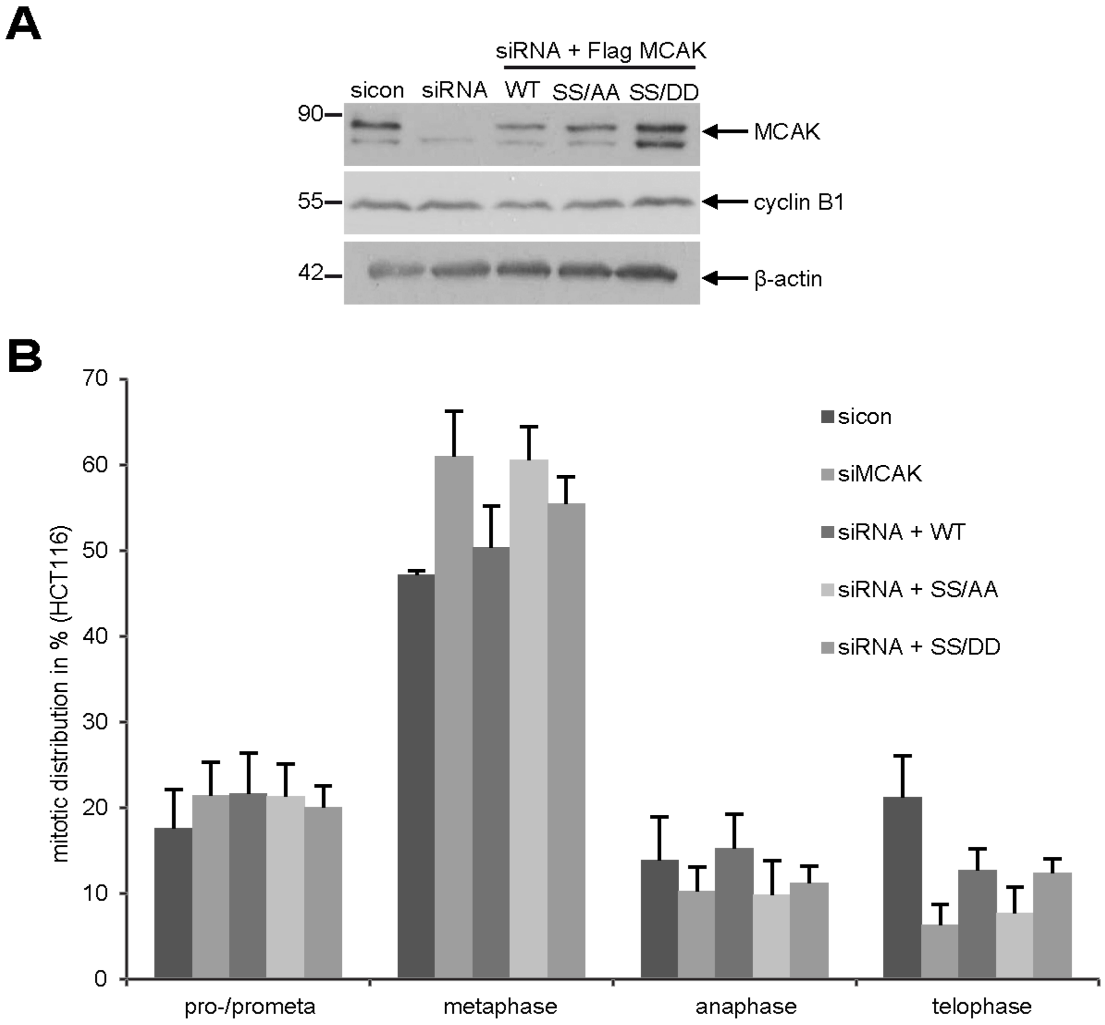

Figure S3: Expression of MCAK SS/AA impacts cell cycle progression and reduces the inter-centromere distance. Rescue experiment: HCT116 cells were transfected with siRNA targeting only endogenous MCAK and followed by the rescue with Flag MCAK wild type and its mutants Flag MCAK SS/AA or Flag MCAK SS/DD. Cells were synchronized to the G2 phase with RO-3306 and released into fresh medium for 1.5 h. (A) Western blot analysis with indicated antibodies for controlling the siRNA efficiency and expression levels of MCAK WT and its mutants. β-actin served as loading control. (B) Transfected HCT 116 cells were stained for α-tubulin and DNA. Analysis of the mitotic sub-phase distribution upon rescue with Flag MCAK and its mutants. The results are displayed as mean ± SD.

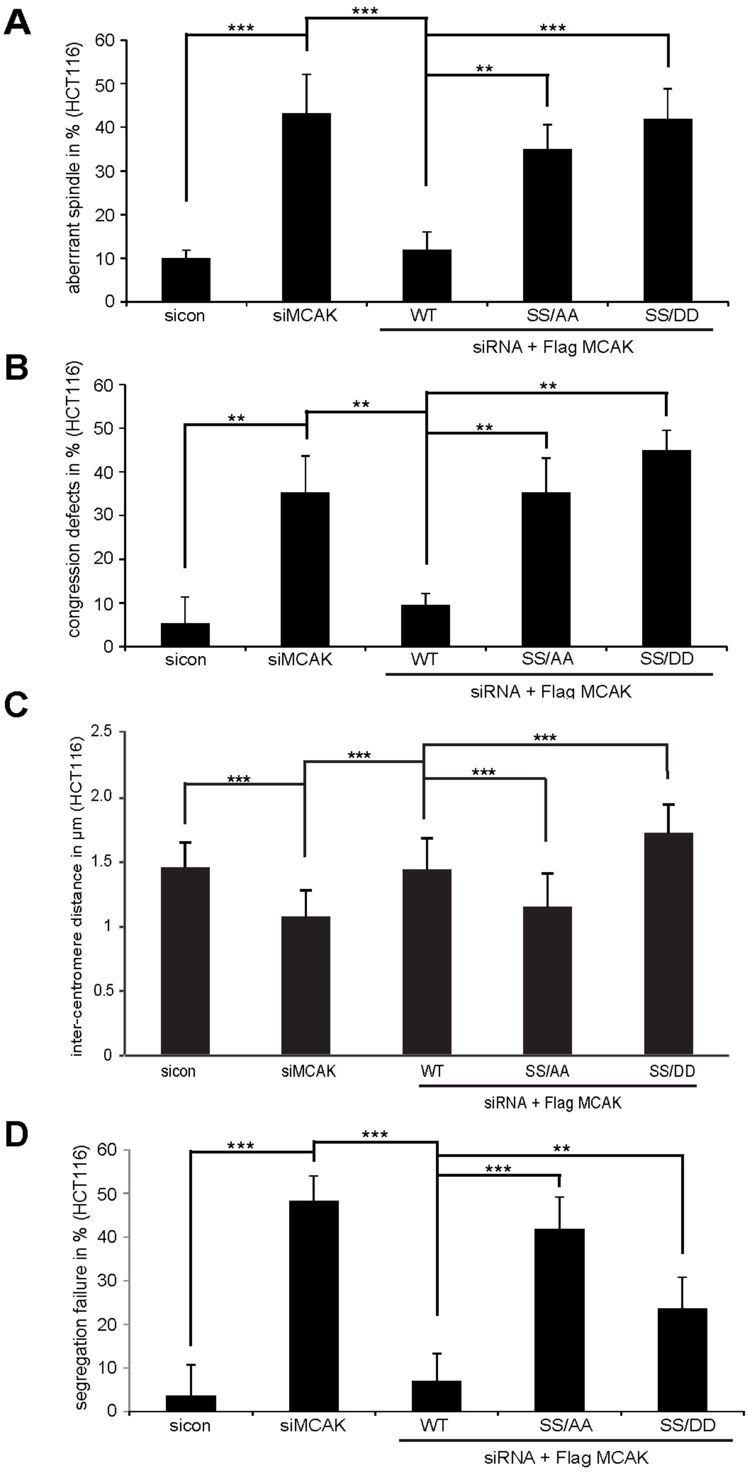

Figure S4: Expression of MCAK mutants generate aberrant spindles as well as cause failures in chromosome alignment and segregation in HCT116 cells. Rescue experiment: HCT116 cells were transfected with siRNA targeting only endogenous MCAK and followed by the rescue with Flag MCAK WT and its mutants. Cells were synchronized to the G2 phase using RO-3306 and released into fresh medium for 1.5 h. Transfected HCT 116 cells were

stained for α-tubulin and DNA. (A) The rate of cells showing defective aberrant spindles. The results are represented as mean ± SD. **$p < 0.01$, ***$p < 0.001$. (B) Percentages of cells displaying failed chromosome positioning. The data are displayed as mean ± SD. **$p < 0.01$. (C) Measurement of the inter-centromere distance in HCT116 cells. HCT116 cells were transfected with Flag MCAK WT or its mutants upon depletion of endogenous MCAK. Cells were synchronized to prometaphase using the Eg5 inhibitor III, the proteasome inhibitor MG132 was provided to the medium containing Eg5 inhibitor for the last hour, and cells were then released into fresh medium with MG132 for 2 h. Cells were then stained for α-tubulin, INCENP and Hec1 as centromere and kinetochore markers. The inter-centromere distance was measured using the LAS AF software. The results are presented as mean ± SD and statistically analyzed. ***$p < 0.001$. (D) The frequency of cells showing defect in chromosome segregation. The results are represented as mean ± SD. **$p < 0.01$, ***$p < 0.001$.
